# Case report: A case of neuro-Behçet's syndrome presenting as brain stem mass lesions

**DOI:** 10.3389/fneur.2023.1218680

**Published:** 2023-07-31

**Authors:** Folusakin Ayoade, Salma Hernandez, Nadine Montreuil, Katherine Drews-Elger, Tanya Quiroz, Candice A. Sternberg

**Affiliations:** ^1^University of Miami Leonard M. Miller School of Medicine, Miami, FL, United States; ^2^Department of Pathology and Laboratory Medicine, Jackson Health System, Miami, FL, United States; ^3^Division of Infectious Disease, Department of Medicine, Jackson Health System, Miami, FL, United States

**Keywords:** neuro-Behçet's, brain stem, uveitis, genital ulcer, immunoglobulin

## Abstract

Neuro-Behçet's syndrome, a severe and rare manifestation of Behçet's disease (BD), can be misdiagnosed due to its challenging clinical presentation. This article presents the case of a 20-year-old cis-gender male with intermittent fever, bilateral uveitis, and neurological symptoms who was found to have multiple brain stem mass lesions on brain imaging. A careful medical history elicited recurrent painful oral and genital ulcerations which were important in making the correct diagnosis. As there are no validated criteria or definite set of tests available to confirm neuro-Behçet's disease, the diagnosis is often established by exclusion after ruling out other potential etiologies. In our case, after an extensive negative workup for infectious, neuro-degenerative and malignant etiologies combined with the patient's medical history, a diagnosis of Behçet's disease with neurological involvement (neuro-Behçet's syndrome) was made. High doses of steroids were given, and the patient had a favorable outcome. Repeated magnetic resonance imaging of the brain 2 years later showed no new brain lesions. Neuro-Behçet's disease should be included as a differential diagnosis of unexplained brain stem lesions in the right clinical context. In these situations, providers should obtain medical histories related to genital and oral ulcers and eye problems as these may help to narrow down the diagnosis. The clinical presentation and challenges of this uncommon presentation of BD including a brief literature review of neuro-Behçet's disease with brain stem mass lesions are discussed in this case study.

## Introduction

Neuro-Behçet's syndrome or disease **(**NBD) is characterized by neurological symptoms in a patient who has suffered or is suffering from other systemic symptoms of Behçet's disease (BD) (1). NBD is relatively rare and has a mean duration of development, of approximately 5 years from the onset of BD (2).

NBD is typically classified into parenchymal or non-parenchymal, based on the area of the brain involved. Parenchymal disease is the most common and includes cerebral, brain stem, or spinal cord disease, while non-parenchymal disease involves the cerebral vasculature, including thrombosis and other stroke syndromes, intracranial hypertension, and meningeal syndromes (2).

Unfortunately, no specific test can isolate the disease, thus making the diagnosis challenging and primarily clinical. Although specific NBD diagnostic criteria have been suggested in the literature, none has been validated (3). The diagnosis of NBD can pose further challenges since it is sometimes difficult to differentiate NBD itself from secondary neurologic symptoms related to BD treatment (2). NBD can cause long-term morbidity and mortality, making early recognition and treatment essential (2).

In this report, we discuss the case of a young immunocompetent male who presented with neurological deficits and ring-enhancing brain stem lesions; a careful medical history revealed oral and genital ulcers and uveitis, leading to the diagnosis of parenchymal NBD. A brief literature review of NBD presenting as a brain mass lesion is also included.

## Case report

A 20-year-old, non-Hispanic Black, cis-gender male with a history of iron deficiency anemia presented with several weeks of intermittent headache and mild fever. While the fever resolved, the headache became constant, and over the following month, he developed a worsened peripheral vision with mild photophobia, left upper extremity weakness, expressive aphasia, and difficulty in walking due to the loss of balance. Since the age of 15, he had experienced recurrent, painful oral and scrotal ulcers that would last for several days at a time. He was sexually active but had not been tested for sexually transmitted infections.

Vital signs were unremarkable. On physical examination, he had left lower facial droop, weakness of his left wrist and fingers, and hyperreflexia of the left lower extremity. A scrotal ulcer was present. On ophthalmologic exam, he had decreased vision in the left homonymous inferior quadrantanopia, bilateral posterior uveitis, chorioretinal scarring, and a single white retinal lesion suspicious for infectious or metastatic disease. On admission, he was found to have CD4 lymphocytopenia (163,000 cells/mcL).

Computerized tomography (CT) of the brain was obtained which showed a heterogenous region with surrounding vasogenic edema centered in the right thalamus and gangliocapsular region (involving the entire right basal ganglia, including the putamen, globus pallidus, and caudate, as well as the posterior aspects of the internal and external capsule). There was a 3-mm leftward midline shift; a mass effect on the Foramen of Monro, right lateral ventricle, and third ventricle; and dilation of the bilateral lateral ventricles. The CT scan also demonstrated a partial effacement of the basal cisterns.

**Figure 1:**
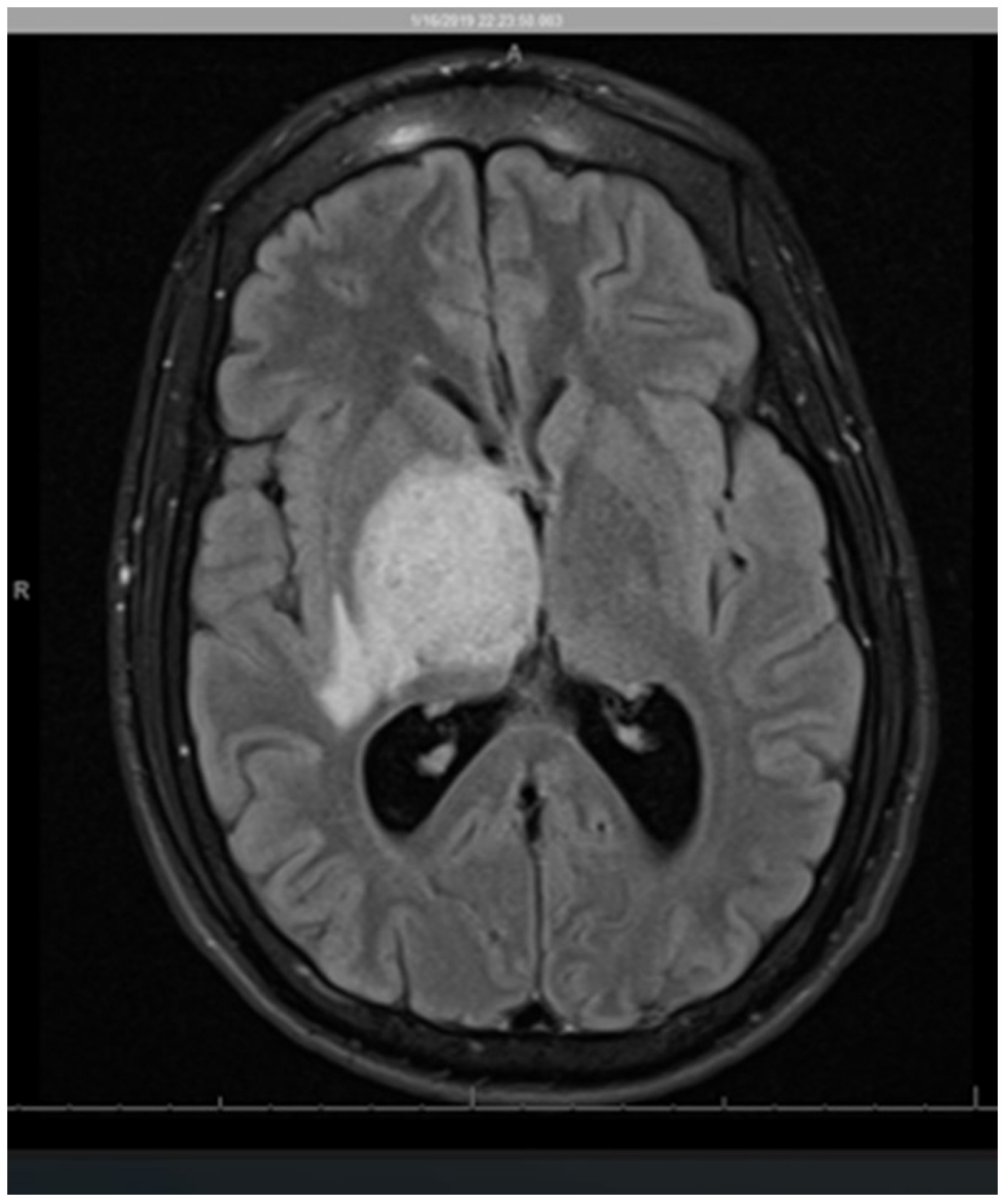
Axial view MR imaging (T2 FLAIR). The image shows a heterogeneous region centered in the right thalamus/gangliocapsular region, involving the entire right basal ganglia, including the putamen, globus pallidus, caudate, and the posterior limb of the internal capsule as well as the posterior aspect of the external capsule. There is a 1-cm right-to-left midline shift and compression of the lateral ventricles and the third ventricle with subsequent mild dilatation of the lateral ventricles concerning obstructive hydrocephalus.

Given the concern for an infectious or neoplastic process or central venous infarct sequelae, contrast-enhanced magnetic resonance imaging (MRI) of the brain was obtained. On MRI, abnormalities were also found involving the right midbrain, with further extension into the pons with mixed regions of T1 isointense and hypointense dense regions and with predominantly T2 and fluid-attenuated inversion recovery (FLAIR) hyperintense lesions. Additionally, there were multiple ring-enhancing lesions in the right thalamic region (with the largest measurement of 9 mm) and midbrain (with the largest measurement of 7 mm), with a surrounding T2 hyperintense region attributable to edema (Figure 1). At this point, additional differential diagnoses were considered, including neurocysticercosis, autoimmune encephalitis, vasculitis, and multiple sclerosis among others.

CT angiography (CTA) of the head and neck with and without contrast showed no large branch arterial occlusion, high-grade stenosis, arteriovenous malformation, or aneurysm.

Due to concerns for autoimmune etiologies, edema, and mass effects, the patient was started on dexamethasone, while the workup was ongoing. Given concerns for an infectious process (brain mass suspicious for brain abscess, in a sexually active person with CD4 lymphopenia), he was also started on broad-spectrum antibiotics.

**Figure 2:**
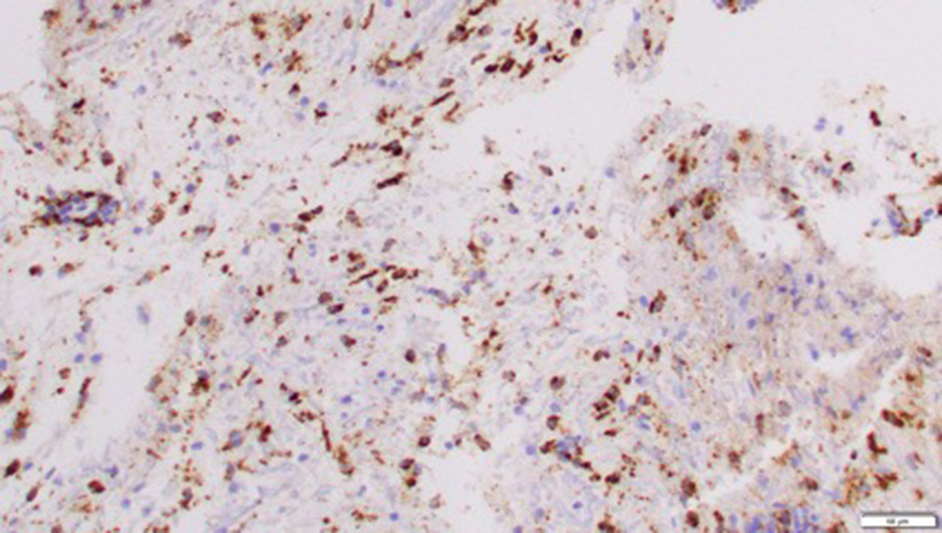
Immunohistochemistry of brain tissue revealed numerous CD68+ histiocytes (Mag. X 20).

Right-sided stereotactic needle biopsy with stealth navigation was performed, and a representative brain tissue sample showed only polymorphonuclear cells on the frozen specimen; therefore, no further neurosurgical intervention was performed. The brain tissue sample histopathology showed parenchymal and perivascular mixed inflammation and reactive gliosis with no evidence of malignancy or inflammation. Infectious studies of the brain tissue biopsy, including cultures (bacterial, fungal, and mycobacterial) and stains (Grocott's methenamine silver, periodic acid—Schiff, and gram stains), were all negative. Most parenchymal and vascular lymphocytes stained positive for CD3, with a subset of these positive for CD8 as well. Immunohistochemistry of the brain tissue also revealed rare CD20+ perivascular cells, numerous CD68+ histiocytes (some of which are microglia), and a Ki67 nuclear proliferation index of 2–3% (Figure 2).

Given the concern for malignancy, a CT scan of the chest, abdomen, and pelvis was completed, but the results were completely unremarkable for any disease process. Other infectious workups included blood cultures and fungal (histoplasmosis and cryptococcus), parasitic (toxoplasmosis and echinococcosis), bacterial (syphilis and tuberculosis), and viral (human immunodeficiency virus, cytomegalovirus, herpes simplex virus, hepatitis B, and hepatitis C) studies, which were all negative.

Further studies revealed that he had mildly elevated antinucleotide antibody titers (1:40—speckled), an erythrocyte sedimentation rate (ESR) of 69 mm/hr (normal range, 0–10), and C-reactive protein (CRP) of 1.3 mg/dl (normal range: <1). He had an elevated total serum protein of 8.3 gm/dL (normal range, 6.3–8.2), a normal serum albumin of 4 gm/dL, an elevated serum globulin of 4.3 gm/dl (normal range, 2–3.5), elevated immunoglobulin (Ig) G of 1830 mg/dl (normal range, 700–1600), and IgA of 534 mg/dl (normal range: 70–400). IgM was within normal limits.

**Figure 3:**
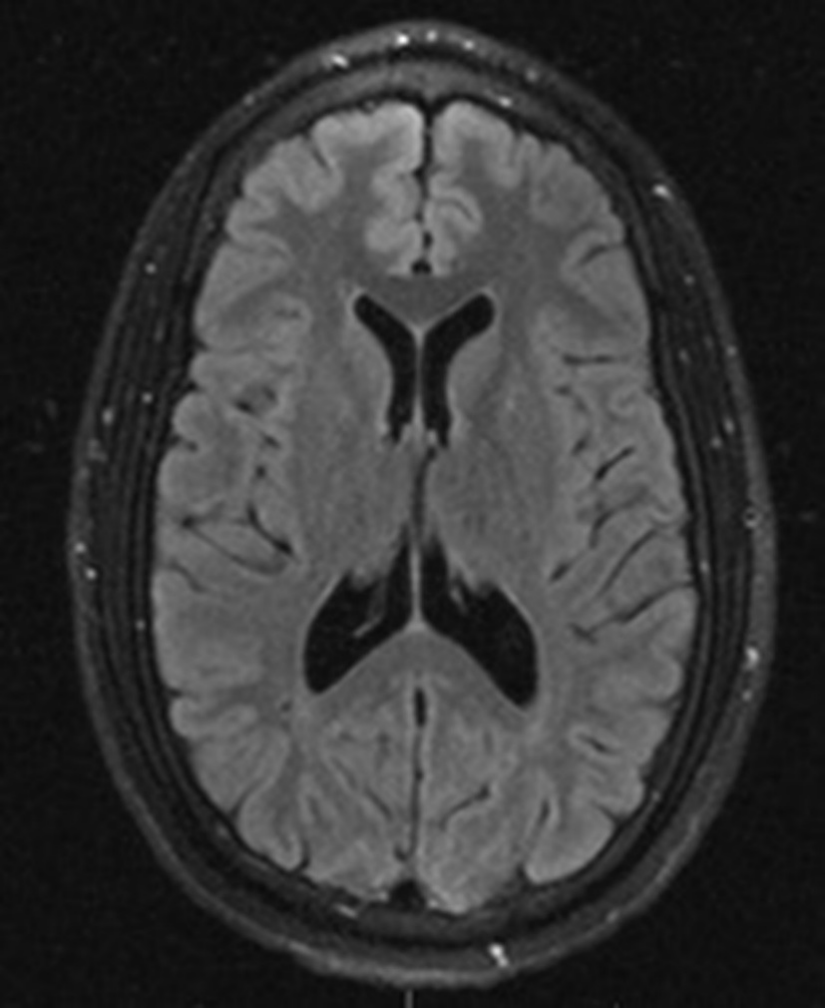
Axial view T2 FLAIR MRI imaging 2 years after initial presentation showed complete resolution of brain lesions.

An MRI of the brain after 5 days of high-dose methylprednisolone (1 g daily) and antibiotics showed an improvement in ring-enhancing lesions of the right thalamus, midbrain, and pons with a less mass effect. His neurological symptoms also improved significantly after several days of treatment. Given the negative infectious workup, antibiotics were discontinued after day 7 of admission. Neuro-Behçet's syndrome was diagnosed, given the patient's history, workup, clinical, and radiographic improvement after steroids and absence of alternate diagnostic etiologies. The pathergy test was negative. Genetic testing for HLA-B51 was collected but not resulted. The patient responded well to high-dose steroids and continued on an oral prednisone taper and azathioprine therapy after discharge. A 2-year follow-up brain MRI later showed no new or enhancing lesions (Figure 3) although the patient continued to have minimal residual deficits in mobility and vision.

## Discussion

Behçet's syndrome (or disease) is a chronic multisystemic inflammatory disorder characterized by systemic vasculitis with perivascular inflammatory infiltrates. The disease is associated with recurrent and relapsing oral ulcers, genital ulcers, skin lesions, eye lesions (notably uveitis), and broader systemic manifestations, such as arthritis and gastrointestinal or central nervous system involvement (4, 5). Behçet's disease (BD) is unique among systemic vasculitides given its ability to affect both arterial and venous circulation and all blood vessel sizes—large, medium, and small.

Approximately 10% of Behçet's syndrome patients have neurologic involvement, which is referred to as neuro-Behçet's disease (NBD). This presentation of the disease is considered a serious manifestation of BD due to the possibility of severe and permanent neurological deficits and associated poor quality of life (6). NBD is more common and severe in men than women, as seen in our study.

In our patient, the clinical presentation of headache, subjective fevers, visual complaints, focal neurological deficit, aphasia, and loss of balance led to initial differential diagnoses that included neurologic malignancy, autoimmune process, and infection. Cerebral CTA did not show any obvious vascular anomalies. Evaluation for the presence of vascular anomalies including cerebral venous thrombosis (CVT) is an integral part of neuro-Behçet's disease (NBD) workup as CVT is considered a major form of presentation of vascular NBD. Lumbar puncture was not obtained in our patient given the mass effect, midline shift, and early decision to pursue stereotactic brain biopsy.

Although the pathergy test was negative, this is not required for diagnosis. A pathergy test is an important diagnostic adjunct for BD or NBD with a specificity of 92% and a sensitivity of 47% in a large series (7). A positive reaction is typically indicated by a papular reaction of ≥2 mm in diameter surrounded by erythema or a pustular reaction of 24–48 h after an obliquely inserted needle prick (7). The brain biopsy performed on the 4th hospital day was negative for malignancy, while all infectious etiology workups, including cultures of brain tissue, were also negative.

Our patient met diagnostic criteria established by the International Study Group for Behçet's disease with a history of recurrent oral and scrotal ulcers over the previous 5 years, self-reported frequent “skin abscesses,” and ocular lesions in the absence of other accountable etiologies (7).

Brain imaging revealed a right basal ganglia/thalamus lesion that was likely causing the patient's left upper extremity weakness. The location of the diencephalon lesion also supported the parenchymal neuro Behçet's diagnosis (8).

The genetic testing result for HLA-B51 was unfortunately not available. HLA-B51 is considered a hallmark of Behçet's syndrome even though its role in pathogenicity is unclear. HLA-B51 may be important in the genetic clustering of Behçet's syndrome (given disease prevalence in eastern Asia and Mediterranean regions) and could play a role in determining clinical phenotypes in this heterogeneous condition (9, 10). On the other hand, the prevalence of HLA-B51 is low in many patients who live in non-endemic regions, suggesting other factors unrelated to HLA-B51 are likely contributing to the pathogenesis of Behçet's disease (4).

Headache is the commonest neurological symptom of neuro-Behçet's disease (NBD). As typified by our case, intermittent headache is commonly reported, up to 82% of BD, with migraine being the commonest type of primary headache (11, 12). Intermittent headache in young patients (40 years or younger) with unexplained brain lesions should prompt further consideration for NBD including a history of oral and genital ulcers and ocular symptoms (13).

The brain stem is the most commonly involved area of the brain. At the same time, 20% of individuals with neurologic symptoms were asymptomatic in one series (6, 14). The varied presentations of neuro-Behçet's disease may mimic other disease processes (3, 6, 8). Coupled with the rarity of the disorder, this often makes obtaining an accurate diagnosis a drawn-out process. In our case, the MRI findings on admission, showing thalamic and midbrain ring-enhancing lesions, in conjunction with the patient's history of recurrent oral/genital lesions and ocular lesions make NBD a possible differential diagnosis (15). Many patients with NBD do not often discuss their recurrent genital and oral ulcers when they present for evaluation, and providers may have to specifically elicit this history to make the correct diagnosis (16). Increased awareness about NBD among providers, especially when unique associations exist, in the absence of alternative diagnoses, could reduce the burden of unnecessary diagnostic testing and treatments (17).

In addition to CD4 lymphocytopenia, our patient's parenchymal and vascular lymphocytes from the biopsied brain tissue mostly expressed CD3, with a subset of these positive for CD8 and rare CD20 cells. The significance of these findings is not entirely clear, but CD3+ CD20+ cells may have a role in autoimmune disease and CD20+ malignancy. A similar abnormal immunophenotypic profile of peripheral lymphocytes has been described in Behçet's disease, including increased absolute numbers of CD4^−^CD8 bright and CD4^+^CD8^+^ cells, reflecting an immune dysregulation as underlying disease pathogenicity (18).

In our patient, in addition to T and B cells, there were numerous microglia/histiocytes and some macrophages. There were no eosinophils, Langerhans cells, or large histiocytic cell infiltrates to suggest histiocytosis such as Rosai–Dorfman disease, Langerhans cell histiocytosis, or Erdheim–Chester disease. The microglia/histiocytes in our case are considered to be reactive and responding to a pathological insult related to BD and not necessarily related to histiocytosis. More studies are needed to understand the association of T-lymphocyte subsets with the pathogenesis of Behçet's disease.

Even though our patient had elevated ESR and CRP, these are very non-specific markers of inflammation that may be evident at presentation but are of limited value in the differential diagnosis of NBD (1).

Our case shared some similarities and a few notable differences when compared to other cases of Neuro-Behçet's disease with brain mass lesions previously published in the literature. A summary of our literature review as of the time of submitting this article is summarized in Table 1.

**Table 1:** Summary of neuro-Behçet's disease with brain stem mass lesions.

**Age/ Gender**	**Brain stem area affected**	**Main clinical presentation**	**HLA-B51 status**	**Pathergy**	**Ig status**	**Histopathology main findings**	**Outcome**	**References**
20^*^ Male	Thalamus, midbrain pons	Headache, photophobia, focal weakness, aphasia	NA	Negative	Elevated IgG and IgA	Perivascular inflammation and reactive gliosis	Resolution of the lesion but minimal deficits in mobility and vision.	Current case
46 Male	Mesencephalon	Left lower limb hemiparesis	NA	Negative	NA	ND	Resolution of symptoms after 10 days	(13)
39 Male	Thalamus	Hemiparesis, hyperreflexia, and ataxic gait	NA	NA	NA	ND	Clinical improvement	(19)
33 Male	Midbrain	Headache, nausea, vomiting	Negative	Positive	WNL	Reactive gliosis without inflammatory cell infiltration	Clinical improvement and shrinkage of lesions	(20)
34 Male	Midbrain and pons	Forgetfulness, irritability, unsteady gait, dysarthria, lower extremity weakness	Positive	Positive	ND	ND	Initial improvement to steroids but died 1 month later due to aspiration	(16)
43 Female	Thalamus, cerebral peduncle	Throbbing headache, photophobia, facial paresis, and ptosis	NA	NA	ND	Extensive gliosis and perivascular cuffing by foamy macrophages	Marked improvement, with complete remission of paresis and ptosis	(21)
47 Male	Pons	Ataxia, dysarthria, hyperreflexia, neurogenic bladder	Positive	ND	ND	ND	Died	(22)
41 Male	Pons	Dysarthria, truncal and limb ataxia, hyperreflexia	Positive	Negative	IgD WNL	ND	Improvement in symptoms and brain lesions	(22)

^*^Index case; BG, basal ganglia; Ig, immunoglobulin; WNL, within normal limits; ND, not done; NA, not available.

All the cases except the two cases described by Hirose et al. had an association with oral or genital ulcers (22). As a group, effective treatment involved the use of high-dose or pulsed steroids.

The role of hyperglobulinemia and hyperimmunoglobulinemia in the pathogenesis of Behçet's disease (BD) is not clear but has been an area of some interest (23, 24). Our patient had elevated serum globulin and immunoglobulins including IgG and IgA. A Mediterranean study comparing 70 BD patients and 35 healthy controls demonstrated a significant elevation in the level of serum globulin (g/dl) in the BD group compared with healthy controls (*p* < 0.001) (23). This hyperproteinemia was explained by an increase in the concentration of specific polyclonal immunoglobulins related to B-cell activation (23). More studies are needed in this area to determine if serum globulin and immunoglobulin could be included as part of a screening tool to determine who may be at additional risk for BD and by extension, NBD when epidemiological and clinical history suggests.

A management option for acute NBD is based on expert opinions as good controlled or comparative trials are lacking (1, 25). Moderate- to high-dose steroids are typically recommended which can be followed by tapering doses of steroids or a prolonged course of colchicine for maintenance (25). If high-dose steroids are ineffective, pulsed steroids or infliximab should be considered. In addition to infliximab, other immunosuppressants and disease-modifying therapies (DMTs) such as azathioprine, methotrexate, mycophenolate, and cyclophosphamide may also be employed in management. These DMTs offer the advantage of providing a steroid-sparing option, reducing the frequency of further neurological relapse, and maintaining prolonged anti-inflammatory effects (1). In our case, we provided an oral prednisone taper and azathioprine therapy after a course of high-dose steroids. In those with CVT, the use of anticoagulation is controversial but still advocated by many experts in addition to immunosuppressants (1).

The limitations of our case include (i) unavailability of HLA-B51 with potential utility in diagnosis as alluded to above and (ii) no CSF analysis done as a spinal tap was avoided due to concern for increased intracranial pressure at presentation. CSF studies have a supportive role in the diagnosis of NBD and are recommended if there are no contraindications (1). Apart from helping to rule out CNS infections, CSF studies may show CSF pleocytosis, elevated CSF protein, and interleukin-6 which are often present in parenchymal, and to a lesser extent, in brain stem NBD (1). CSF pleocytosis has been shown to portend a poor prognosis in NBD (6). Normal CSF parameters do not, however, rule out a diagnosis of NBD and IL-6, which in particular may not always correlate with disease activity (26).

In conclusion, providers should consider neuro-Behçet's disease as a possible differential diagnosis in brain stem lesions, especially in young adults with genital or oral lesions. The role of T-lymphocyte subsets, hyperglobulinemia, and hyperimmunoglobulinemia in Behçet's disease should be better defined.

## Data availability statement

The original contributions presented in the study are included in the article/supplementary material, further inquiries can be directed to the corresponding author.

## Ethics statement

Written informed consent was obtained from the individual(s) for the publication of any potentially identifiable images or data included in this article.

## Author contributions

FA contributed to the conception and original draft of the manuscript. FA and SH contributed to the discussion section. NM contributed to writing the introduction and abstract sections. TQ contributed to preparing the radiographic images. KD-E contributed to the generation of the histopathological slide. CS contributed to editing the whole manuscript. CS and FA were involved in the critical revision of the manuscript. FA and TQ were involved in the direct patient care of the subject described in the case report. All authors contributed to the manuscript revision and read and approved the submitted version.
